# The Rogan-Gladen estimator for outcome misclassification

**DOI:** 10.1093/aje/kwag057

**Published:** 2026-07-08

**Authors:** Jessie K. Edwards, Paul N. Zivich, Bonnie E. Shook-Sa, Stephen R. Cole

**Affiliations:** 1 Department of Epidemiology, University of North Carolina at Chapel Hill, Chapel Hill, NC; 2 Nuffield Department of Population Health, University of Oxford, Oxford, UK

## Abstract

Outcome measurement error is common in epidemiologic studies and often leads to bias in estimates of prevalences, risks, and their contrasts. The Rogan-Gladen estimator (1) provides an elegant solution to account for misclassification of a binary outcome when estimating risk or prevalence, which, in turn, can be used to produce valid estimates of contrasts in these measures. Here, we review the Rogan-Gladen estimator, provide intuition for its properties, and briefly illustrate how it can be combined with approaches to account for other sources of bias.

## Rogan-Gladen Estimator

Say the parameter of interest is the risk or prevalence of disease *ρ* = *P*(*Y* = 1). However, *Y* was not observed in our study. Instead, our study captured an error-prone measure Y*. Sensitivity (*α*) is the probability of detecting a true event using the error-prone measure, *α* = *P*(Y* = 1∣*Y* = 1). Specificity (*β*) is the probability of correctly identifying a non-event, *β* = *P*(Y* = 0∣*Y* = 0).

The parameter of interest *ρ* can be estimated by the Rogan-Gladen estimator, which is a function of ρ* = *P*(Y* = 1), *α*, and *β*,

$$\hat{\rho }=\frac{{\hat{\rho }}^{*}+\beta -1}{\alpha +\beta -1}.$$

To build intuition, consider that observed prevalence can be written as a function of the true prevalence, sensitivity, and specificity using the law of total probability such that

$$P\left(Y^{*}=1\right)=P\left(Y^{*}=1|Y=1\right)P(Y=1)+P\left(Y^{*}=1|Y=0\right)P(Y=0)$$

which can be written in our above notation as ρ* = *αρ* + (1 − *β*)(1 − *ρ*). From this expression for ρ*, we can solve for *ρ* as follows:

(1) $$\begin{array}{c} \rho^{*}=\alpha \rho +(1-\beta )(1-\rho )\\ \rho^{*}=\alpha \rho +1-\rho -\beta +\beta \rho \\ \rho^{*}=\rho (\alpha +\beta -1)+(1-\beta )\\ \frac{\rho^{*}}{\left(\alpha +\beta -1\right)}=\rho +\frac{1-\beta }{\left(\alpha +\beta -1\right)}\\ \rho =\frac{\rho^{*}+\beta -1}{\alpha +\beta -1}. \end{array}$$

Thus, the Rogan-Gladen is a plug-in estimator based on the mismeasured proportion, sensitivity, and specificity.

Note that the Rogan-Gladen estimator fails if *α* = 1 − *β* because the denominator is 0. Intuitively, the Rogan-Gladen estimator cannot provide information about the true prevalence if the measurement is exactly as likely to produce a positive result for true cases as for true noncases. When *α* = 1 − β, ρ* = *α* = 1 − *β* regardless of true prevalence (Equation 1), implying that ρ*, *α*, and *β* provide no information about the true prevalence.

In their 1978 paper, Rogan and Gladen argue that a process is only a “test” if it detects disease better than chance, such that *α* > 1 − *β*. When *α* > 1 − *β*, the positive predictive value *P*(*Y* = 1∣Y* = 1) = *αρ*/ρ* is greater than *ρ*. However, the Rogan-Gladen estimator *can* be used to account for outcome misclassification induced by processes that are not tests by this definition. Indeed, if testing positive is negatively associated with true disease status, the estimator still effectively leverages sensitivity and specificity to estimate *ρ* (Appendix S1, Figure S1).

**Figure 1:**
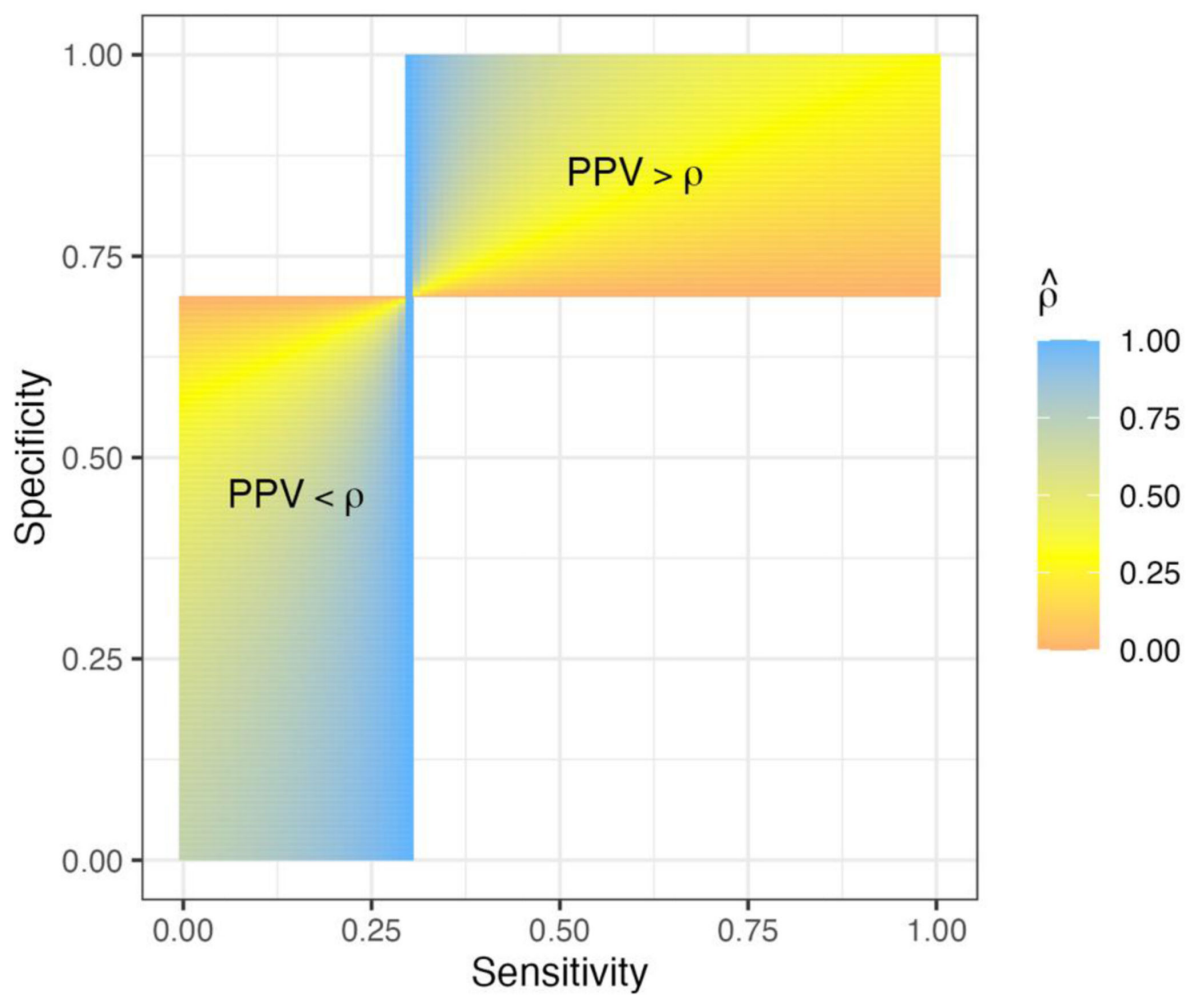
Estimated prevalence using Rogan-Gladen estimator under various combinations of sensitivity and specificity for an observed prevalence of 30%. Shaded regions denote combinations of sensitivity and specificity that are compatible with the observed prevalence. The top right rectangle contains values of sensitivity and specificity for which the positive predictive value PPV is greater than prevalence. These are the settings where the test performs better than chance. The lower left rectangle represents combinations of sensitivity and specificity where PPV is less than prevalence. These are the settings where the test performs worse than chance. Nonshaded regions are combinations of sensitivity and specificity incompatible with the observed prevalence of 30%. For example, if the observed prevalence is 30% and specificity is above 0.7, sensitivity must be at least 0.3. PPV: Positive predictive value

Applying the Rogan-Gladen estimator with combinations of sensitivity and specificity that are incompatible with observed prevalence ρ* will yield $\hat{\rho }<0\;$ or $\hat{\rho }>1$ (Appendix S2). Such incompatibility occurs when the observed value ρ* could not have arisen from the chosen combination of sensitivity and specificity for any possible value of *ρ* and thus provides constraints on possible combinations of sensitivity and specificity. Figure 1 presents estimates $\hat{\rho }$ for a setting with ρ* = 30% under various combinations of sensitivity and specificity. The nonshaded regions of the figure are areas where sensitivity and specificity are incompatible with ρ*. Figure S2 extends Figure 1 for multiple values of p*.

## Information About Sensitivity and Specificity

Sensitivity and specificity are rarely known with certainty. Typically, researchers estimate these parameters using validation data, which may be internal to the main study or external. In this setting, we plug in estimates of sensitivity and specificity based on the validation data ($\hat{\alpha }\;$ and $\hat{\beta }$, respectively), such that the Rogan-Gladen estimator is

$$\tilde{\rho }=\frac{{\hat{\rho }}^{*}+\hat{\beta }-1}{\hat{\alpha }+\hat{\beta }-1}.$$

With an appropriately sampled validation study (2), a simple estimator of sensitivity is the proportion who have a detected outcome Y* = 1 among the *m* participants in the validation study with *Y* = 1. A simple estimator of specificity is, among the *r* participants in the validation study with *Y* = 0, the proportion with detected outcome Y* = 0.

## Variance Estimation

As described by the original Rogan-Gladen paper, if sensitivity and specificity are known, a closed-form variance estimator for $\hat{\rho }\;$ is

$$\hat{Var}(\hat{\rho })=\frac{{\hat{\rho }}^{*}\left(1-{\hat{\rho }}^{*}\right)}{n{\left(\alpha +\beta -1\right)}^{2}}.$$

where *n* is the sample size of the main study. When sensitivity and specificity are estimated, the closed form of the variance for $\tilde{\rho }$ provided by Rogan and Gladen is

$$\hat{Var}(\tilde{\rho })=\frac{{\hat{\rho }}^{*}\left(1-{\hat{\rho }}^{*}\right)}{n{\left(\hat{\alpha }+\hat{\beta }-1\right)}^{2}}+\frac{\hat{\alpha }(1-\hat{\alpha }){\tilde{p}}^{2}}{m{\left(\hat{\alpha }+\hat{\beta }-1\right)}^{2}}+\frac{\hat{\beta }(1-\hat{\beta }){\left(1-\tilde{p}\right)}^{2}}{r{\left(\hat{\alpha }+\hat{\beta }-1\right)}^{2}},$$

where the extra terms convey the uncertainty due to estimation of sensitivity and specificity (3).

Variance of $\hat{\rho }\;$ and $\tilde{\rho }$ may also be estimated using the empirical sandwich variance estimator (4). Alternatively, a version of the nonparametric bootstrap in which both main study and validation data are resampled may be used in this setting (5). Note that, when *α* is near 1 − *β*, the variance can become large regardless of the chosen variance estimator.

## Integrating the Rogan-Gladen into Epidemiologic Analyses

Outcome misclassification is rarely the only bias present in epidemiologic studies. The Rogan-Gladen estimator can be used in combination with standardization to account for other biases, e.g., selection bias due to informative missing data. If misclassification is independent of these other sources of bias, *ρ* can be estimated by standardizing ρ* to the desired distribution of covariates and then use this standardized prevalence estimate in the expression for $\hat{\rho }\;$ or $\tilde{\rho }$. If misclassification is differential by a covariate in the standardization set described above, sensitivity and specificity must be allowed to vary by level of this covariate, as described in Appendix S3.

Often, epidemiologists are interested in estimating the risk difference by comparing proportions between groups: *ρ*(*A* = 1) − *ρ*(*A* = 0), where *ρ*(*A* = *a*) = *P*(*Y* = 1∣*A* = *a*). In this setting, the Rogan-Gladen estimator can be applied within each stratum of exposure, i.e.,

$$\tilde{\rho }(a)=\frac{{\hat{\rho }}^{*}(a)+\hat{\beta }(a)-1}{\hat{\alpha }(a)+\hat{\beta }(a)-1},$$

where ${\hat{\rho }}^{*}(a)$ is an estimator for *P*(Y* = 1∣*A* = *a*) and $\hat{\alpha }(a)\;$ and $\hat{\beta }(a)$ are the estimates of sensitivity and specificity among participants with *A* = *a*. In special cases where sensitivity and specificity are assumed to be *nondifferential* with respect to both *A* and covariates, overall estimates of sensitivity and specificity may be used such that $\hat{\alpha }(a)=\hat{\alpha }\;$ and $\hat{\beta }(a)=\hat{\beta }$ for all values of *a*. If confounding is present, estimator $\tilde{\rho }(a)$ may be extended as noted in Appendix S3.

## Example

We illustrate the Rogan-Gladen approach to estimate the prevalence of HIV among pregnant participants in the East Africa Cross Border Integrated Health Study, where participants completed a survey and a rapid HIV test (6).

Of the 244 pregnant participants, 225 agreed to complete the HIV rapid test. Of those, 12 tested positive, yielding an estimated prevalence of 5.3%. However, rapid tests may have imperfect sensitivity and specificity. Chetty et al (7) conducted a validation study of the Determine HIV 1/2 Ag/Ab Combo, a rapid test commonly used in the region during this time period, among pregnant people (Table 1, Figure S3). We used these validation data to account for the imperfect performance of the rapid test using the Rogan-Gladen estimator. Data and code for the example in R and Python are available at https://github.com/edwardsjk/publication-code/tree/main/RoganGladen.

Using these validation data, estimated sensitivity was 208 / (208+31) = 0.941 and estimated specificity was 31 / (31+1) = 0.969. Applying these values and our naïve prevalence estimate $\left({\hat{\rho }}^{*}=0.053\right)$, our estimate of *ρ* was

$$\tilde{\rho }=\frac{0.053+0.969-1}{0.941+0.969-1}=0.024=2.4\text{\%}.$$

Using the variance estimator described above, the estimated variance was

$$\begin{array}{c} \hat{Var}(\tilde{\rho })=\frac{0.053(1-0.053)}{225{\left(0.941+0.969-1\right)}^{2}}+\frac{0.941(1-0.941)0.024^{2}}{221{\left(0.941+0.969-1\right)}^{2}}\\ +\frac{0.969(1-0.969){\left(1-0.024\right)}^{2}}{32{\left(0.941+0.969-1\right)}^{2}}=0.0014, \end{array}$$

for a standard error of $\sqrt{0.0014}=0.037$.

### Combining with approaches to account for missing data

Recall that 19 of the 244 survey participants (8%) refused the HIV rapid test. Participants with missing HIV test results were, on average, younger, more highly educated, less likely to drink alcohol, and more likely to report exchanging sex for cash. To account for informative missing data, we used inverse probability of participation weights. Specifically, under the assumption that misclassification was independent of these variables, we reweighted participants with available rapid test results in the main study data to have the same distribution of covariates as the entire study sample. Weights were 1/*P*(*S* = 1 ∣ *L*), where *S* indicates an observed HIV rapid test result and *L* is the vector of the 4 covariates mentioned above. The weighted prevalence of HIV according to the rapid test in the main study data was 5.4%. After applying the Rogan-Gladen estimator, the estimated prevalence of HIV in the main study was

$$\hat{\rho }=\frac{0.054+0.969-1}{0.941+0.969-1}=0.025=2.5\text{\%}.$$

Because weights were used, the standard variance estimator was no longer appropriate. We used M-estimation to implement the empirical sandwich variance estimator, and the estimated standard error was 0.037 (Appendix S4).

## Conclusions

The Rogan-Gladen estimator is a simple and powerful tool to account for outcome misclassification. Yet measurement error is overwhelmingly ignored in epidemiologic studies (8). Here, we have addressed two potential reasons for the reticence to account for measurement error: 1) difficulty with uncertainty quantification; and 2) the need to combine misclassification corrections with approaches to handle other sources of bias. However, perhaps the largest barrier to use of the Rogan-Gladen estimator is lack of appropriate information about sensitivity and specificity.

For valid inference, the Rogan-Gladen estimator requires estimates of sensitivity and specificity that are transportable to the main study. Without validation data, one may parameterize the Rogan-Gladen estimator using beliefs about sensitivity and specificity or in sensitivity analyses under a range of plausible values of these parameters. As seen in Appendix S2, some combinations of sensitivity and specificity are incompatible with observed prevalence, which may arise when knowledge that informed the choice of these parameter values is flawed or validation data are not transportable to the study sample. In sensitivity analyses, these values of sensitivity and specificity should not be considered.

## Supplementary Material

Supplement

## Figures and Tables

**Table 1:** Summary of validation data from Chetty et al. proving counts positive by rapid test and by the gold standard (HIV enzyme immunoassay)

	Positive by Rapid Test	Negative by Rapid Test
Gold standard positive	208	13
Gold standard negative	1	31

## Data Availability

Data used in the example are available at https://github.com/edwardsjk/publication-code/tree/main/RoganGladen
