# Supplementary material for: The Rogan-Gladen estimator for outcome misclassification
Source: Am J Epidemiol. Author manuscript; Available in PMC 2026 Jun 16. (PMC7619182; doi:10.1093/aje/kwag057)
Supplement: Supplement [file EMS212865-supplement-Supplement.docx]

Supplemental material

The Rogan-Gladen estimator for outcome misclassification

Jessie K. Edwards, Paul N. Zivich, Bonnie E. Shook-Sa, and Stephen R. Cole ^1^

**Included Material**

[Appendix S1. Illustration that the Rogan-Gladen estimator produces valid results even when $\boldsymbol{\alpha}+\boldsymbol{\beta}<\boldsymbol{1}$ 2](#_Toc223939667)

[Figure S1 2](#_Toc223939668)

[Appendix S2. Compatibility of sensitivity, specificity, and observed prevalence 3](#_Toc223939669)

[Figure S2 4](#_Toc223939670)

[Appendix S3: Differential misclassification 5](#_Toc223939671)

[Appendix S4. Example Details 6](#_Toc223939672)

[Figure S3 7](#_Toc223939673)

## Appendix S1. Illustration that the Rogan-Gladen estimator produces valid results even when $\boldsymbol{\alpha}\boldsymbol{+}\boldsymbol{\beta}\boldsymbol{<}\boldsymbol{1}$

In Figure S1 below, we show panels for true prevalence ranging from 10% to 90%. For each point corresponding to a combination of sensitivity and specificity in each figure, we computed the observed prevalence. Next, we used that observed prevalence and the Rogan-Gladen estimator to account for the misclassification. For all values except those where $\alpha=1-\beta$, the Rogan-Gladen estimate matched the true prevalence, illustrating that the Rogan-Gladen works well even when $\alpha+\beta<1$.

Figure S1. Prevalence estimated using the Rogan-Gladen estimator (colors) for true prevalence values ranging from 10% to 90% (panels) in settings with sensitivity (x-axis) and specificity (y-axis) ranging from 0 to 1. The white diagonal line through running from top left to bottom right of the figure illustrates points where $a=1-\beta$ where no answer can be computed. Aside from this line, the Rogan-Gladen estimator estimates prevalence without bias for all values of sensitivity and specificity.


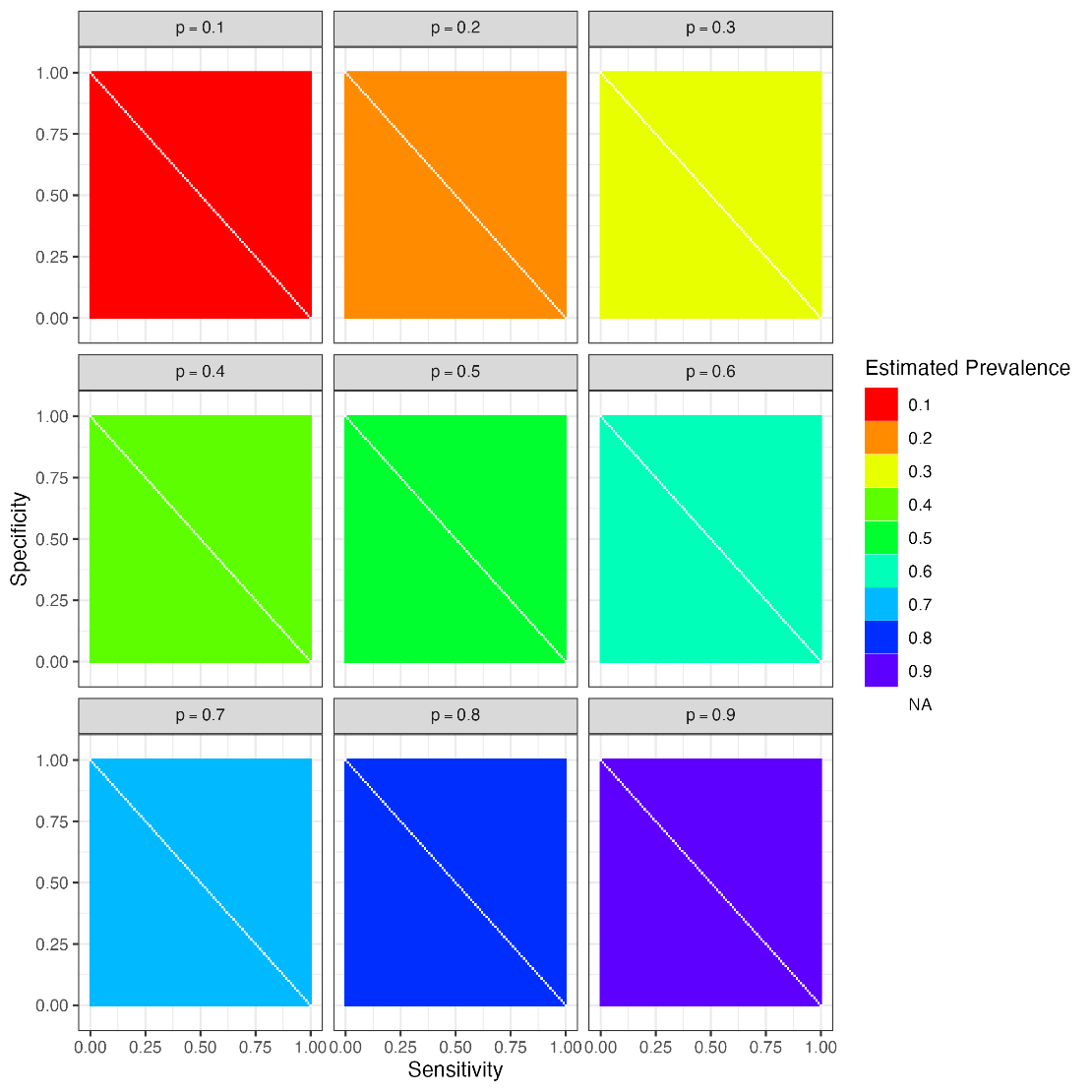


## Appendix S2. Compatibility of sensitivity, specificity, and observed prevalence

Figure S2 is an extended version of Figure 1 in the main paper that illustrates the difference between observed prevalence and prevalence estimated using Rogan-Gladen estimator $\hat{\rho}$ for 9 values of observed prevalence ranging from 0.1 to 0.9 applying values of sensitivity and specificity ranging from 0 to 1. Estimated prevalence values below 0 and above 1 have been deleted to illustrate the compatible values of sensitivity, specificity, and observed prevalence. For each observed prevalence value, the plot has 2 compatible regions: the top right square or rectangle, where $\alpha>1-\beta$ and $\alpha>\rho^{*}$ is where the positive predictive value of the test is greater than $\rho$. This is where the test works better than chance. The lower left rectangle also includes compatible values, but for these values of $\alpha$and $\beta$, the positive predictive value is less than $\rho$, implying that the test works worse than chance. However, as seen in the main text, the Rogan-Gladen estimator still yields accurate results in settings with positive predictive values lower than $\rho$ (or, equivalently, $\alpha+\beta<1$), as long as the selected values of $\alpha$ and $\beta$ are compatible with the observed prevalence.

Figure S2: Difference between observed prevalence and prevalence estimated using Rogan-Gladen estimator (“bias”) for 9 values of observed prevalence ranging from 0.1 to 0.9 applying values of sensitivity and specificity ranging from 0 to 1.


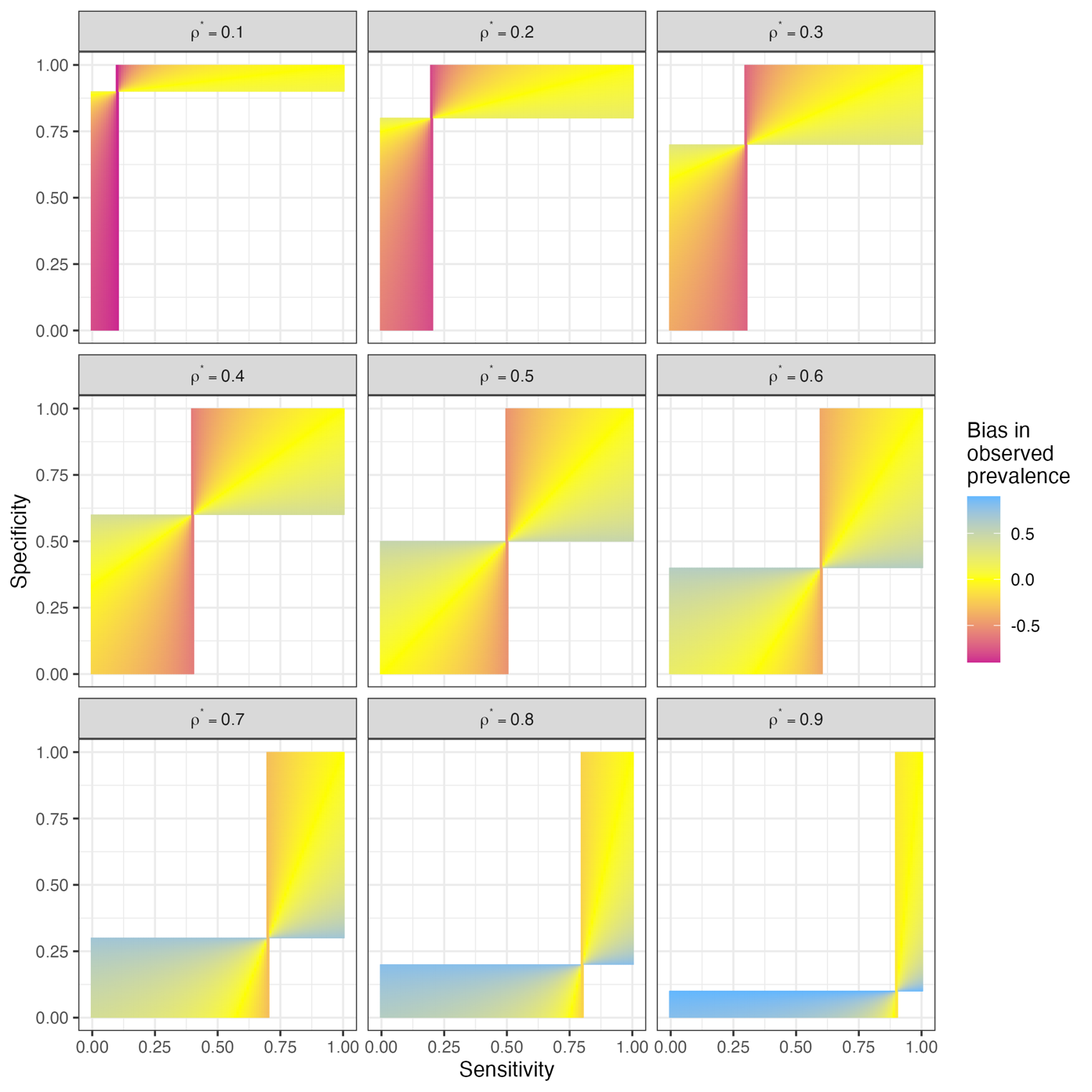


## Appendix S3: Differential misclassification

To obtain a consistent estimate of risk or prevalence, $\hat{\alpha}$ and $\hat{\beta}$ must be computed in a validation study that reflects the target population for the main study (1). If the covariate distribution in the validation study differs from the target population and $\alpha$ and $\beta$ vary by levels of these covariates, the following algorithm may be used: 1) compute sensitivity and specificity within strata of covariates: $\hat{\alpha}(L)$ and $\hat{\beta}(L)$. 2) Apply the Rogan-Gladen estimator with $\hat{\alpha}(L)$, $\hat{\beta}(L)$, and $\hat{\rho}^{*}\left( L \right)=\hat{P}(Y^{*}=1|L)$ to compute stratum specific risk or prevalence estimates $\hat{\rho}(L)$. 3) Standardize $\hat{p}(L)$ across levels of $L$ in the study sample to compute $\hat{\rho}$: $\hat{\rho}=\sum_{l} \hat{\rho}\left( L=l \right)P(L=l).$

If $\alpha$ and $\beta$ vary by a covariate $Z$ that is involved in another source of bias (e.g., confounding), a simple approach is to apply the Rogan-Gladen estimator within each stratum of $Z$ and then standardize across levels of $Z$. For example, if misclassification is differential by $Z$ and $Z$ is a confounder, the following algorithm could be applied: 1) compute sensitivity and specificity within strata of $Z$; 2) estimate $\rho^{*}(a,z)$, the predicted probability of $Y^{*}$ within strata of $A$ and $Z$; 3) Apply the Rogan-Gladen within levels of $a$ and $z$, such that $\hat{\rho}\left( a,z \right)=\frac{\rho^{*}\left( a,z \right)+\beta\left( a,z \right)-1}{\alpha\left( a,z \right)+\beta\left( a,z \right)-1}$; 4) standardize across levels of L: $\hat{\rho}(a)=\sum_{l} \hat{\rho}\left( a,Z=z \right)P(Z=z)$.

In settings where misclassification varies by a confounder and sensitivity and specificity do not naively transport from the validation data conditional on exposure and confounder, alternative approaches are needed, as described by Ross et al (2).

## Appendix S4. Example Details

**Analysis using M-estimation**

In the example, we repeated the analysis using M-estimation (3), which provided the empirical sandwich estimate of the variance. To implement this approach, we stacked together the outcome indicator $Y^{*}$ for the $n$ records in main study data with $Y^{*}$ from $m$ records in the validation data with $Y=1$ and $Y^{*}$ from the $r$ records in the validation data with $Y=0$. In the stacked data we created a new variable $V$ with values 0 if the record was from the main study, 1 if it was one of the $m$ records in the validation study with $Y=1$, and 2 if it was one of the $r$records from the validation study with $Y=0$. Letting $X=(V, Y^{*})$ and $\theta=(\alpha,\beta, \rho^{*}, \rho)$, the stacked estimating function was

$$g_{\theta}\left( X;\theta\right)=\left[ \begin{aligned} g_{\alpha}\left( X;\alpha\right) \\ g_{\beta}\left( X;\beta\right) \\ g_{\rho^{*}}\left( X;\rho^{*} \right) \\ g_{\rho}(X;\alpha, \beta, \rho^{*}, \rho) \end{aligned} \right]= \left[ \begin{aligned} I(V=1)(Y^{*}-\alpha) \\ I(V=2)({[1-Y}^{*}]-\beta) \\ I(V=0)(Y^{*}-\rho^{*}) \\ \rho\left( \alpha+\beta-1 \right)-(\rho^{*}+\beta-1) \end{aligned} \right].$$

The asymptotic variance of $\theta$ was estimated via the empirical sandwich estimator $E\{B^{-1}M\left( B^{-1} \right)^{T}\}$, where $B=E\{g^{'}\left( X;\theta\right)\}$, $g^{'}\left( X;\theta\right)=-\left\{ \frac{\partial g\left( X;\theta\right)}{\partial\theta} \right\}$, and $M=E\{g\left( X;\theta\right)g\left( X;\theta\right)^{T}\}$. Using this approach, the estimated prevalence $\hat{\rho}$ was 2.4% (95% CI: 0.0, 9.7; standard error: 3.7%). An introduction to M-estimation is presented by Ross et al. (4)

*M-estimation with missing data*

To account for the missing data in addition to the misclassification, we again stacked together the outcome indicator $Y^{*}$ for the $n$ records in main study data with $Y^{*}$ from $m$ records in the validation data with $Y=1$ and $Y^{*}$ from the $r$ records in the validation data with $Y=0$. In the stacked data we created a new variable $V$ with values 0 if the record was from the main study, 1 if it was one of the $m$ records in the validation study with $Y=1$, and 2 if it was one of the $r$records from the validation study with $Y=0$. We additionally created a matrix $W$ with $n+m+r$rows corresponding to study participants in the main study (with and without missing data on $Y^{*}$) and validation study. $S$ was an indicator of nonmissing outcome data in the main study (and was set to 0 for participants in the validation study). Columns were covariates age, sex work, alcohol use, and education level. All covariates were set to 0 for participants in the validation study (where covariates were not measured. Let $\pi$ represent the probability of having a nonmissing outcome given covariates. With $X=(V, Y^{*}, W, S)$ and $\theta=(\alpha,\beta, {\pi, \rho}^{*}, \rho)$, the stacked estimating function was

$$g_{\theta}\left( X;\theta\right)=\left[ \begin{aligned} g_{\alpha}\left( X;\alpha\right) \\ g_{\beta}\left( X;\beta\right) \\ g_{\pi}(X;\pi) \\ g_{\rho^{*}}\left( X; \rho^{*}, \pi\right) \\ g_{\rho}(X; \alpha, \beta, \rho^{*}, \rho) \end{aligned} \right]= \left[ \begin{aligned} I(V=1)(Y^{*}-\alpha) \\ I(V=2)({[1-Y}^{*}]-\beta) \\ I\left( V=0 \right)X(S-\pi) \\ I\left( V=0 \right)S\left( Y^{*}-\rho^{*} \right)\pi^{-1} \\ \rho\left( \alpha+\beta-1 \right)-(\rho^{*}+\beta-1) \end{aligned} \right].$$

Using this approach, the estimated prevalence was 2.5% (standard error 0.037).

Figure S3**:** Estimated prevalence using Rogan-Gladen estimator under various combinations of sensitivity and specificity for the example’s observed prevalence of 5.3%. Shaded regions denote combinations of sensitivity and specificity that are compatible with the observed prevalence. The top right rectangle contains values of sensitivity and specificity for which the positive predictive value PPV is greater than prevalence. These are the settings where the test performs better than chance. The lower left rectangle represents combinations of sensitivity and specificity where PPV is less than prevalence. These are the settings where the test performs worse than chance. Nonshaded regions are combinations of sensitivity and specificity incompatible with the observed prevalence of 5.3%. The point represents values of sensitivity and specificity from the validation data, which are compatible with the observed prevalence.


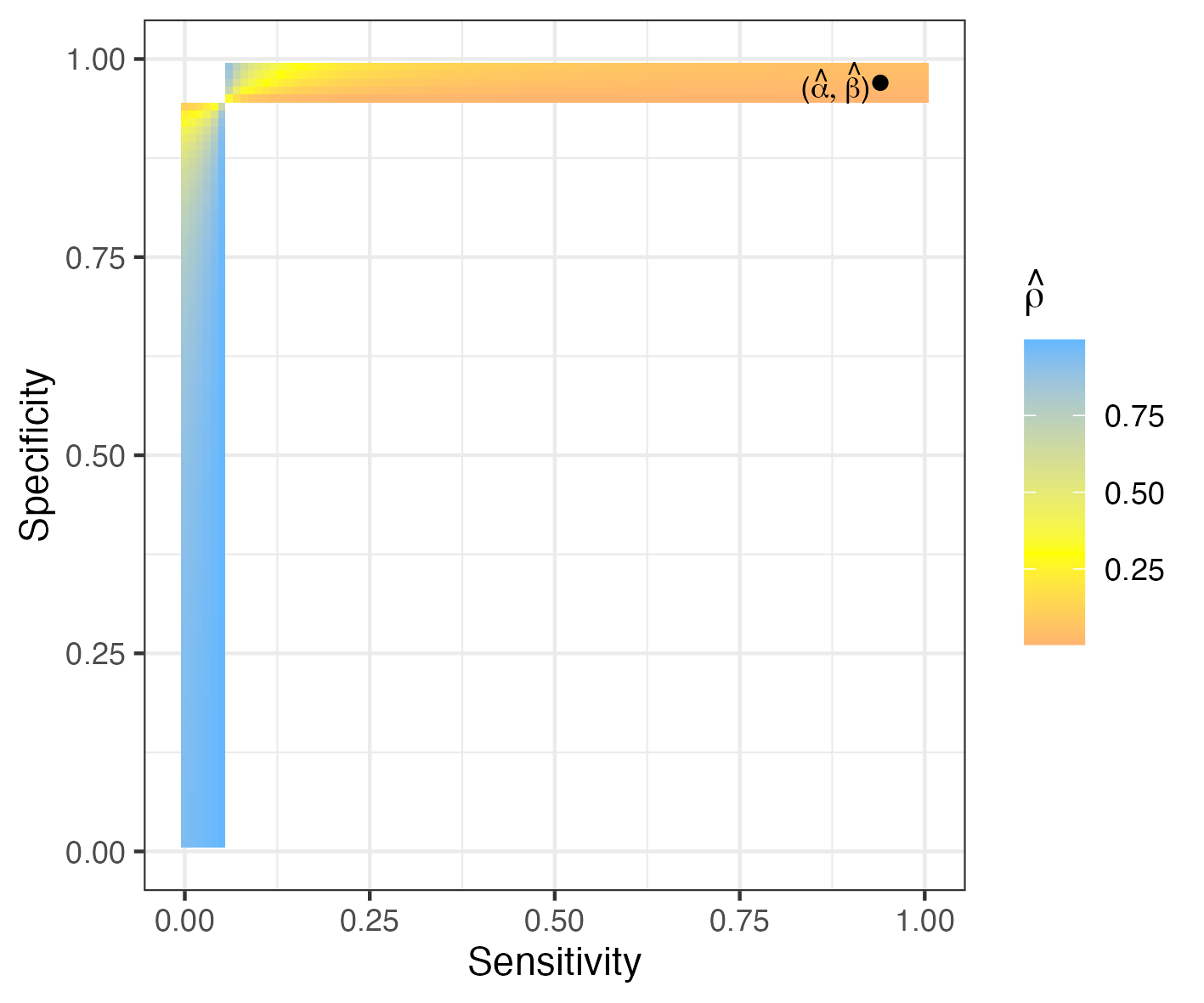


1. Edwards JK, Cole SR, Shook-Sa BE, et al. When Does Differential Outcome Misclassification Matter for Estimating Prevalence? *Epidemiology*. 2023;34(2):192.

2. Ross RK, Cole SR, Edwards JK, et al. Leveraging External Validation Data: The Challenges of Transporting Measurement Error Parameters. *Epidemiology*. 2024;35(2):196.

3. Stefanski LA, Boos DD. The Calculus of M-Estimation. *Am. Stat.* 2002;56(1).

4. Ross RK, Zivich PN, Stringer JSA, et al. M-estimation for common epidemiological measures: introduction and applied examples. *Int. J. Epidemiol.* 2024;53(2):dyae030.
